# A randomized trial evaluating the utility of non-targeted biopsies for colorectal neoplasia detection in adults with inflammatory bowel disease: a pilot study protocol

**DOI:** 10.1186/s40814-023-01434-8

**Published:** 2024-02-01

**Authors:** Sanjay K. Murthy, Luba Marderfeld, Dean Fergusson, Tim Ramsay, Charles N. Bernstein, Geoffrey C. Nguyen, Vipul Jairath, Robert Riddell

**Affiliations:** 1 Department of Medicine, University of Ottawa, Ottawa, ON Canada; 2 The Ottawa Hospital, IBD Centre, Ottawa, ON Canada; 3 Clinical Epidemiology Program, Ottawa Hospital Research Institute, Ottawa, ON Canada; 4 School of Epidemiology and Public Health, Faculty of Medicine, University of Ottawa, Ottawa, ON Canada; 5 Department of Medicine, Max Rady College of Medicine, Rady Faculty of Health Sciences, University of Manitoba, Winnipeg, MB Canada; 6 University of Manitoba IBD Clinical and Research Centre, Winnipeg, MB Canada; 7 Mount Sinai Centre for Inflammatory Bowel Disease, University of Toronto, Toronto, ON Canada; 8 Institute for Health Policy, Management and Evaluation, University of Toronto, Toronto, ON Canada; 9 Department of Medicine, Western University, London, Canada; 10 Department of Epidemiology and Biostatistics, Western University, London, Canada; 11 Department of Pathology, Mount Sinai Hospital, Toronto, ON Canada

**Keywords:** Inflammatory bowel disease, Ulcerative colitis, Crohn’s disease, Colorectal cancer, Neoplasia, Dysplasia, Colonoscopy, Screening, Random biopsy

## Abstract

**Background:**

Persons with inflammatory bowel diseases are at increased risk of developing colorectal cancer and require frequent colonoscopy surveillance. Guidelines recommend taking 30 to 40 non-targeted biopsies throughout the colorectum to detect “invisible” neoplasia in this setting, despite a lack of evidence supporting this practice. We sought to assess the utility of this practice through a randomized controlled trial. We first propose an internal pilot study to assess recruitment potential, protocol adherence and data capture to guide the full trial.

**Methods:**

We have designed a multi-centre, parallel-group, non-inferiority randomized controlled trial to test the utility of non-targeted biopsies as an adjunct to colonoscopy surveillance for neoplasia detection in persons with inflammatory bowel disease involving the colorectum in routine clinical practice. Participants are randomized 1:1, stratified by study site, to either standard of care high-definition white-light colonoscopy with 32 to 40 non-targeted biopsies of non-neoplastic-appearing mucosa along with a sampling of abnormal-appearing mucosa (control group) or modified colonoscopy with targeted sampling alone (intervention group). The primary outcome for the full trial will be the proportion of persons with ≥ 1 neoplastic focus detected during colonoscopy. For the pilot phase, we will assess the feasibility of recruiting a minimum of 15% of the estimated sample size within 1 year, under identical conditions as the full trial, while maintaining ≥ 90–95% rate of protocol adherence and data capture. These participants will contribute data to the full trial. The trial is being conducted at 12 centres across Canada, with a total sample size of 1952 persons.

**Discussions:**

The trial protocol has been approved by the ethics committees of all participating sites, and the pilot study has received funding through the Canadian Institutes of Health Research (PJT 159607). If feasibility metrics are met during the pilot phase, we will complete the full trial. The trial outcomes will contribute to update the practice guidelines in this area.

**Trial registration:**

ClinicalTrials.gov, NCT04067778.

**Supplementary Information:**

The online version contains supplementary material available at 10.1186/s40814-023-01434-8.

## Background

Inflammatory bowel diseases (IBD), including Crohn’s disease (CD) and ulcerative colitis (UC), are characterized by acute and chronic inflammation of the gastrointestinal tract and extra-intestinal organs and are associated with substantial morbidity related to active disease symptoms, bowel surgery and colorectal cancer (CRC). IBD afflicts more than 0.3% of persons in developed nations, and the incidence is rising in newly industrialized countries [1–3]. CRC is a devastating complication of IBD involving the colorectum, accounting for as much as 15% of IBD-related deaths [4]. Persons with colorectal IBD have a 1.5- to 3-fold higher risk of developing CRC relative to age-matched members of the general population [5–7] and require frequent screening with colonoscopy to detect and treat pre-cancers and early-stage cancers [5–7].

Guidelines have long recommended that screening exams include 33 or more non-targeted (“random”) biopsies throughout the colorectum to screen for “invisible” neoplastic lesions, in addition to targeted biopsies or resection of visible lesions [8–11], based on mathematical modelling in one study showing that ≥ 33 jumbo forceps biopsies are required throughout the colorectum to detect one neoplastic focus with 90% confidence in an individual with pancolitis [12]. The rationale for taking non-targeted biopsies is based on a fear of “invisible” neoplasia (dysplasia) in persons with colorectal IBD, emanating from molecular studies reporting widespread DNA damage in areas of chronic colitis (“field carcinogenesis”) [12–15], as well as reports of high rates of synchronous and metachronous CRC in persons with colorectal IBD who have neoplasia identified during screening colonoscopy [16–25]. Furthermore, neoplasia in IBD can take on unconventional growth patterns, including flatter growth and growth resembling acute and chronic inflammatory changes (nodular, stricturing and ulcerated growth patterns) [26, 27] that could easily evade detection during colonoscopy.

Despite the theoretical value of taking non-targeted biopsies in this setting, there is an absence of controlled data to support this practice. To date, only one small randomized controlled trial (RCT) from Japan has addressed this question and demonstrated the non-inferiority of eliminating non-targeted biopsies [28]. Importantly, most of the aforementioned studies suggesting increased risks of invisible neoplasia were conducted during eras of limited treatment options for IBD, poorer resolution endoscopes (even optical endoscopes) and relative absence of endoscopy practice quality parameters, all of which could have contributed to the challenges in properly identifying neoplasms during colonoscopy. The past two decades have witnessed the introduction of highly effective biologic therapies for treating IBD [29–34], a shift from symptom-based treatment to targeting complete bowel healing [35–40], vast improvements in endoscope technology [41–43] and introduction of numerous endoscopy practice quality standards (bowel preparation quality, cecal intubation rates, colon inspection times and polyp detection rates) [44–47], all of which have likely contributed to reduced rates of CRC and improved detection of subtle and indistinct lesions during colonoscopy in this population [48, 49]. Recent studies have shown that more than 90% of neoplastic lesions are visible using high-definition white-light endoscopy (HD-WLE) [50], that non-targeted biopsies of normal appearing mucosa have an exceedingly low yield for detecting neoplasia (0.1–0.2% of biopsies) [50, 51] and that the rates of synchronous and metachronous cancers in the setting of neoplasia have declined considerably over time [51, 52]. Additionally, the prognostic significance of minute foci of neoplasia detected only through non-targeted biopsies is unclear. These factors have led many experts to question the value of continuing to take widespread non-targeted biopsies for neoplasia screening [51, 53, 54].

On the other side of the argument, several recent large observational studies have reported that non-targeted biopsies continue to identify up to 20% of neoplastic foci in colorectal IBD. While the extent to which non-targeted biopsies in these retrospective reviews were taken from the areas of subtle mucosal abnormality, active inflammation or poor bowel preparation (where the view was obscured), as opposed to normal-appearing mucosa, is uncertain, these reports have nonetheless fueled the debate regarding this practice. Notably, non-targeted biopsies were most useful in individuals with other CRC risk factors in these studies, including primary sclerosing cholangitis, prior colorectal neoplasia, active inflammation and extensive colonic scarring [50, 51, 55].

In a recent survey of Canadian gastroenterologists, 55% of respondents stated that non-targeted biopsies are not an effective method for neoplasia detection in IBD patients; yet, more than 75% of respondents reported that they still routinely carry out this practice [56]. Amongst panellists from the Surveillance for Colorectal Endoscopic Neoplasia Detection and Management in Inflammatory Bowel Disease Patients (SCENIC) international consortium, 45% agreed and 30% disagreed with the practice of performing non-targeted biopsies when using HD-WLE [50]. The variability in physicians’ perceptions regarding this practice may be a combination of the evolving and inconsistent data on this topic, the persistence of this recommendation in guidelines and the absence of definitive evidence refuting the merits of this practice.

In addition to uncertainty regarding its effectiveness, reliance on non-targeted biopsies for neoplasia detection can detract from careful inspection of the colorectum and reduce the likelihood of identifying visible abnormalities [28, 57]. Extensive biopsy sampling may also increase the risk of significant colonic bleeding and bowel perforation, particularly in light of a growing demographic of elderly IBD patients [58] and increasing use of anti-platelet and anti-coagulant agents in society [59, 60]. Moreover, this practice adds 15 to 20 min to colonoscopy time [56, 57], which reduces procedural capacity and increases wait times for other patients. Finally, the specimen processing and pathologist costs associated with obtaining and interpreting 30 to 40 biopsy specimens account for up to 50% of the cost of performing a colonoscopy in this setting [61], placing potentially unnecessary financial strain on the health care system.

These arguments provided a strong impetus to conduct a well-powered RCT to evaluate the utility of non-targeted biopsies as an adjunctive intervention during screening and surveillance colonoscopy in persons with colorectal IBD.

## Study objectives

The primary aims for the pilot phase of the study are to assess recruitment feasibility, protocol adherence and data capture. The overarching study aim is to evaluate whether the practice of taking routine interval non-targeted biopsies could be eliminated from colonoscopy screening and surveillance in persons with colorectal IBD without impacting the overall neoplasia detection rate. Additional aims for the full trial are to assess the impact of eliminating non-targeted biopsies on the rates of visible neoplasia, missed invisible neoplasia, adverse events and future CRC, as well as on procedure time.

## Study eligibility

All adults (≥ 18 years old) with colorectal IBD who are undergoing routine surveillance colonoscopy and meet the following criteria will be included:

- Minimum 8 years colorectal IBD duration or any duration if concomitant PSC
- Minimum historical endoscopic or histologic disease extent of proctosigmoiditis (UC) or 1/3 colorectum (CD)
- At least 50% of colorectum present and meeting the minimum criteria for disease extent
- In symptomatic remission at the time of colonoscopy (Harvey-Bradshaw Index < 5 in CD [62] or partial Mayo Score (pMayo) ≤ 2 in UC [63])
- Colonoscopy being performed using high-definition white-light endoscopy
- Minimum of 1 year since the last colonoscopy performed for neoplasia surveillance
- Complete colorectal examination
- Good to excellent bowel preparation after washing (Boston Bowel Preparation Scale score of 2 to 3 in all segments [64])
- Limited inflammatory activity— ≤ 25% of colorectum affected by moderate-to-severe inflammation (pMayo 2–3 in UC or non-aphthous ulceration in CD) or no more than 50% of colorectum affected by mild inflammation (Mayo 1 in UC or aphthous inflammation in CD)

The following are the exclusion criteria:

- Unable to provide informed consent
- History of colorectal cancer
- Prior subtotal or total colectomy (> 50% of colon removed)
- Undergoing colonoscopy for repeat evaluation of recently identified colorectal neoplasia
- Undergoing pancolonic dye spray chromoendoscopy (DCE) or virtual chromoendoscopy (VCE)

## Trial design and conduct

This is a multi-centre, parallel-group, non-inferiority RCT. Participants meeting the eligibility criteria will be randomly allocated to one of the two groups, stratified by study site, using a web-based central randomization scheme coordinated by the Ottawa Methods Centre:

- Control group—standard of care screening/surveillance colonoscopy, with 32–40 interval non-targeted biopsies of non-neoplastic-appearing mucosa, as well as targeted biopsies or endoscopic resection of suspicious visible mucosal abnormalities
- Intervention group—modified screening/surveillance, with targeted biopsies or endoscopic resection of suspicious visible mucosal abnormalities alone

Randomization will occur intra-procedurally, once all clinical and endoscopic eligibility criteria have been confirmed. Study investigators and/or their trained research assistants will perform the following tasks: (i) assess study eligibility and obtain informed consent from patients; (ii) directly observe the study-related colonoscopy (trained assistant), to confirm intra-procedural eligibility criteria, randomize patients, ensure colonoscopy protocol adherence and obtain procedure-related information pertinent for the trial; (iii) perform medical record review, to collect historical and post-procedural data, including histology of biopsy/resection samples; (iv) conduct a virtual interview with patients a minimum of 2 weeks following the study colonoscopy, to ascertain procedure-related adverse events; (v) record all study-related data in a web-based case report form (developed by the Ottawa Methods Centre); and (vi) facilitate transfer of de-identified physical or electronic slide sets from patients with neoplasia/dysplasia for central adjudication.

Aside from the number of non-targeted biopsies taken, endoscopists will be permitted to practice within acceptable standards of care, in keeping with a pragmatic trial. The sampling and specimen collection methodology will be performed as per investigators’ practices. A minimum of 10-min colonoscopy withdrawal time will be mandated in both groups, inclusive of mucosal sampling time, to limit the potential wide differential in mucosal inspection time for neoplastic lesions between the two groups. For patients who have previously undergone partial colectomy, a minimum withdrawal time of 6 min will be mandated in both groups and a minimum of 20 non-targeted biopsies will be mandated in the control group. Of note, the current standards for colonoscopy withdrawal time to optimize neoplasia detection in the non-IBD screening population are a minimum time of 6 min [65] and a preferred time of 10 min [66]. Further, up to 10 interval non-targeted biopsies will be allowed for histologic disease assessment in the intervention group as part of routine clinical care.

Pathological specimens will be initially reviewed by site pathologists. In patients with one or more samples graded as neoplasia or dysplasia (definite or indefinite), a representative set of histologic samples will be centrally reviewed and graded by two expert IBD pathologists (RHR, JC). Inter-observer agreement amongst pathologists is reported to be much poorer for dysplastic samples as compared to normal mucosal samples [67, 68]. Where the site and central interpretations differ, the central adjudication will take precedence and override the site-specific interpretation. Where the two central adjudicators disagree, a consensus agreement amongst pathologists at the central adjudicating site will be sought.

## Study setting

This study will take place at 12 centres across Canada:

- The Ottawa Hospital, University of Ottawa (lead site)
- Mount Sinai Hospital, University of Toronto
- London Health Sciences Centre, Western University
- McMaster University Health Centre
- Thunder Bay Regional Health Sciences Centre, Northern Ontario School of Medicine
- University Health Sciences Centre, Memorial University of Newfoundland
- McGill University Health Centre
- Health Sciences Centre, University of Manitoba
- St. Paul’s Hospital, University of British Columbia
- Pacific Digestive Health/Royal Jubilee Hospital, University of Victoria
- Nova Scotia Health Authority, Dalhousie University
- University of Alberta Hospitals

## Study outcomes

The following are the pilot phase outcomes (minimum metrics to justify full trial):

(i) Randomization of ≥ 15% of full trial sample size (292 participants) within 1 year of study initiation across the participating sites
(ii)  < 10% rate of major protocol violations, on a per-patient basis
(iii)  < 5% miss rate for non-essential variables and < 1% for essential variables (group allocation, intra-procedural interventions and neoplasia findings) on a per-patient basis
(iv)  < 5% loss-to-follow-up (LFU) for the 2-week post-procedural assessment

### Future trial outcomes

The primary outcome is the proportion of persons with ≥ 1 neoplastic focus in the colorectum.

The following are the secondary outcomes:

(i) The mean and median number of neoplastic lesions per person
(ii) The rate of advanced neoplasia (any of CRC, high-grade neoplasia, large neoplasia (> 2 cm diameter) or multifocal neoplasia (≥ 3 independent neoplastic foci throughout the colorectum))
(iii) The neoplasia yield of non-targeted biopsies
(iv) The mean and median number of tissue samples per person
(v) The mean and median procedure time
(vi) Rate of serious adverse events (SAE) within 2 weeks of colonoscopy [69] (hospital admission, bowel perforation, severe rectal bleeding requiring blood transfusion and/or repeat colonoscopy, acute cardiac or respiratory compromise or death)
(vii) Proportion of persons referred for colectomy based on neoplastic findings
(viii) The mean and median time to the next recommended surveillance examination
(ix) Incidence of CRC over 5 years following study colonoscopy (obtained through linkage of patient data to provincial cancer registries or direct patient contact, at least 5 years following study completion)

We will conduct exploratory sub-group analyses of the primary outcome and multiple secondary outcomes based on disease type (CD vs UC), disease duration and prior biologic exposure.

## Neoplasia definitions

Definitions and classifications for neoplasia in this trial were developed through consensus amongst steering committee members. Neoplastic foci identified in non-targeted biopsies will be treated as unique lesions, under the assumption that non-targeted biopsies are taken at a sufficient distance from one another so as to not sample the same lesion. Conversely, multiple targeted biopsies of a dysplastic area, or endoscopic resection of a visible lesion, will be counted as a single neoplastic focus. Furthermore, all histologic types and grades of neoplasia identified during colonoscopy will be counted for the primary analysis, including all low-grade and high-grade “adenomas” (including all tubular, villous, tubulo-villous and serrated designations), “sessile serrated lesions”, unspecified neoplasia or dysplasia and CRC. While the magnitude of risk may differ across neoplastic lesions, such differences are not easily quantifiable. Adopting simplified study definitions was important to produce clear and objective measures of neoplasia rates. As an exploratory analysis, the rate of advanced neoplasia (CRC, high-grade neoplasia) in the two groups will be evaluated separately.

Only *definite* for neoplasia or dysplasia (low-grade, high-grade or CRC) will contribute towards the outcome assessment; foci that are *indefinite* for neoplasia or dysplasia (i.e. having histologic criteria that are suggestive of neoplasia but inconclusive) will not count towards outcome assessment due to uncertainty of diagnosis [70] but will be reviewed centrally and reported with the study findings. Slide sets from participants with one or more samples graded as neoplasia/dysplasia (definite or indefinite) by site pathologists will have a representative set of all histologic samples reviewed centrally by two expert IBD pathologists (RHR, JC). Disagreements between the central pathologists will be rectified through consensus.

## Study variables

The study case report form, detailing all variables that are being captured as part of this trial, is provided in Additional file 1.

## Sample size calculation

The estimated sample size to assess non-inferiority of the intervention in the full trial is 1952 persons (976 per group), based on a 1-sided significance level of 2.5% and 80% power, assuming neoplasia detection rates of 15% and 14.5% in our control and intervention groups, respectively, and a non-inferiority margin of 5%. Our sample size estimate for the pilot phase (≥ 15% of the study sample size recruited within 1 year of study initiation) is guided by the feasibility of completing the full trial within an acceptable time frame of 5 to 7 years.

Our estimates for neoplasia yield in the 2 groups were guided by pooled analyses of clinical trials and observational cohorts conducted by the SCENIC consortium [50]. The pooled estimate from 4 studies (382 patients) of HD-WLE with non-targeted and targeted sampling was 17%, while the pooled estimate from 7 studies (1289 patients) of DCE (an alternative detection method to non-targeted sampling) was 13.6% [50]. A reasonable estimate for neoplasia detection rate in the control group is thus 15%. The pooled estimates for the proportion of persons diagnosed with neoplasia in non-targeted biopsies alone in these analyses ranged from 1.2% (in studies using DCE) to 1.5% (in studies using HD-WLE). However, we anticipated that these would be overestimates of the difference that would be observed in a present-day RCT, given continued improvements in IBD treatments, endoscope technology and colonoscopy practice quality, as well as a propensity towards closer inspection for visible lesions in the absence of non-targeted biopsies [28, 57]. Therefore, we estimated a 0.5% reduction in the neoplasia detection rate without non-targeted biopsies for this trial.

The proposed non-inferiority margin of 5% is based on a large meta-analysis of 14 surveillance cohort studies in 671 patients with colonic IBD diagnosed with low-grade neoplasia (LGN) by either non-targeted or targeted biopsies, which calculated a pooled rate of progression of LGN to CRC of 0.8% per year [52]. Based on this estimate, if one-third of persons with LGN were missed in our intervention group relative to our reference group (i.e. reduction in absolute neoplasia detection rate from 15 to 10%), it would result in a theoretical 0.04% increased risk per year of CRC in our intervention group or 1 in 500 persons over 5 years. The steering committee and content experts deemed this to be an acceptable upper limit for a reduction in the neoplasia detection rate for the trial, given the potential for reduced procedural risks and costs with avoidance of non-targeted biopsies. This estimate also aligns with the only other RCT on this topic, in which investigators used a non-inferiority margin equivalent to roughly one-third of the baseline neoplasia detection rate [28].

## Data analysis

We will evaluate the pilot study results based on participant recruitment rate, protocol adherence and quality of data capture. Treatment allocation will remain concealed to study investigators following the completion of the pilot phase so as to not influence ongoing study recruitment. We require a minimum of 292 participants (> 15% of the full trial sample) to be recruited within 1 year of trial initiation at each site to deem adequate feasibility of recruitment for the full trial. We will accept up to a 5% rate of major protocol violations, up to a 1% miss rate of major variables and up to a 5% LFU amongst study participants recruited during the pilot phase. If these metrics are met and there are no safety concerns with the study intervention based on the Data and Safety Monitoring Committee (DSMC) review, we will proceed with recruitment to the full trial.

The full trial results will be assessed by per-protocol analysis and intent-to-treat (ITT) analyses. The chi-square test will be used to analyze the differences in binary categorical outcomes, and Student’s *t*-test will be used to analyze the differences in continuous outcomes. Non-inferiority of the primary outcome (proportion of persons with neoplasia detected) will be demonstrated if the upper limit of a one-sided 97.5% confidence interval around the expected true difference of 0.5% in favour of the control group (15% vs 14.5%) excludes a difference of more than 5%. If the baseline characteristics potentially influencing event rates have a standardized difference of ≥ 0.1, multivariable analysis will be further conducted.

## Trial management

The trial steering committee comprised experts in IBD, cancer epidemiology and trial methodology (SKM, DF, CNB, RHR, GCN, VJ). The steering committee was responsible for the trial design and consensus study definitions. The steering committee members will meet biannually for the duration of the trial to review trial progress. The DSMC comprised three experts in clinical trial methodology who are not directly involved with the trial design or conduct (Dr. Bill Cameron, University of Ottawa; Dr. Tim Ramsay, University of Ottawa; and Dr. Chris Ma, University of Calgary). The DSMC will meet twice yearly to review the trial data and progress and make recommendations regarding the trial continuation and protocol adjustments. A project manager (PM) will oversee all study operations across all participating sites and will liaise closely with the study PI to ensure smooth trial progress. PM tasks will include site initiation and closeout; oversight of patient recruitment, data collection and entry and protocol deviations; training and support of site coordinators; organization and attendance at all study-related meetings; audits of site-specific study data; ensuring the integrity of the electronic data capture tool; coordinating logistics around shipping biopsy specimen samples for central review; and coordinating study site reimbursement. The Canadian IBD Research Consortium will provide in-kind support through a study administrator to assist the PM and PI as well as through engagement of its physician members to optimize trial participation. The Ottawa Methods Centre has developed and maintains the web-based central randomization tool and electronic data capture system that is accessible to each site at the point of care. Only de-identified data is entered into the case report forms (CRFs). CRFs are electronically stored on an encrypted Ottawa Hospital server. Analysts at the Ottawa Methods Centre who are blinded to patient allocation will conduct all study analyses and provide aggregate results to DSMB and study investigators, as required. Study investigators will not have access to the source data or patient allocation until the final analysis for the definitive trial is complete.

## Ethics and dissemination

Each institution has provided Research Ethics Board approval to conduct the study locally. There are no ethical or safety concerns relating to this trial. With the support of the Canadian IBD Research Consortium, Crohn’s and Colitis Canada and the Canadian Association of Gastroenterology, we will widely disseminate the trial results through conference presentations and press releases and will further publish the trial results in peer-reviewed publications and practice guideline updates. As the first well-powered trial on this topic, the study findings are expected to influence clinical practice guidelines worldwide and to be widely cited.

## Discussion

In summary, we will test the feasibility to conduct an adequately powered RCT testing the utility and safety of taking interval non-targeted biopsies to detect neoplasia during colonoscopy screening and/or surveillance in persons with colorectal IBD. In the context of RCT, an initial pilot phase carried out in a specific set of centres enables the establishment and evaluation of essential trial procedures prior to expanding to the complete trial. If successful according to the proposed progression criteria for internal pilot studies [71], we will complete the full trial, from which the findings will inform clinical practice and will be used to update practice guidelines in this area. The overall and pilot study findings will also provide an update to older literature from the pre-biologic era regarding neoplasia rates and the safety of non-targeted biopsies in this setting.

### Supplementary Information

**Additional file 1.** CRF_IBD Neoplasia Surveillance RCT PILOT.

## Data Availability

All data generated or analysed during this study are included in this published article and its supplementary information files.

## Abbreviations

CD: Crohn’s disease
CRC: Colorectal cancer
CRF: Case report form
DCE: Dye spray chromoendoscopy
DSMC: Data and Safety Monitoring Committee
GCP: Good Clinical Practice
HBI: Harvey-Bradshaw Index
IBD: Inflammatory bowel disease
ICF: Informed consent form
LFU: Loss to follow-up
LGN: Low-grade neoplasia
PI: Principal investigator
RCT: Randomized controlled trial
REB: Research Ethics Board
SES-CD: Simplified-Endoscopy Score-Crohn’s Disease
SOPs: Standard operating procedures
UC: Ulcerative colitis
UCEIS: Ulcerative Colitis Endoscopic Index of Severity
VCE: Virtual chromoendoscopy
HD-WLE: High-definition white-light endoscopy
