# Supplementary material for: A randomized trial evaluating the utility of non-targeted biopsies for colorectal neoplasia detection in adults with inflammatory bowel disease: a pilot study protocol
Source: Pilot Feasibility Stud. 2024 Feb 1;10:20. doi: 10.1186/s40814-023-01434-8 (PMC10832187; doi:10.1186/s40814-023-01434-8)
Supplement: Supplementary file 1 — Additional file 1. CRF_IBD Neoplasia Surveillance RCT PILOT. [file 40814_2023_1434_MOESM1_ESM.pdf]

Appendix A
CRF_IBD Neoplasia Surveillance RCT PILOT **(IBD-DYSPLASIA)**

Participant ID: ☐☐ ☐☐☐

BASELINE VARIABLES WORKSHEET	
Sex: Male ◯ Female ◯	Age: (years)
Height: (cm)	Weight: (cm)
Disease Type: (circle one) CD / UC / IBDU	
Duration of IBD: (months) _____	
Date of last colonoscopy (YYYY)	
Maximal histologic extent of colonic involvement (historical)	
Maximal colitis severity based on physician impression (historical)	
History of colorectal dysplasia/adenoma? YES / NO	
Family history of colorectal cancer? YES/NO	
History of PSC	
Smoking history: {Current heavy > ½ PPD/Current light ≤1/2 PPD/ Past Heavy Past Light/social/rare and never}	
Current of past 5'ASA therapy? YES / NO	

Participant ID: ☐☐ ☐☐☐

Total Duration of 5'-ASA therapy (months):
Current or past use of immunosuppressive therapy (azathioprine, 6-MP, methotrexate, biologic, small molecule)?
Past IBD Treatments (specify as many as required from list):<br>Oral 5'ASA, Rectal 5'-ASA, Oral/IV corticosteroids, Cyclosporine/tacrolimis, azathioprine/6-MP, Methotrexate, Anti-TNF therapy, Anti-integrin therapy, Anti-IL-23 therapy, JAK inhibitor, Other biologic/targeted therapy, specify, NONE
Current IBD Treatments (specify as many as required from list below):<br>Oral 5'ASA, Rectal 5'-ASA, Oral/IV corticosteroids, Cyclosporine/tacrolimis, azathioprine/6-MP, Methotrexate, Anti-TNF therapy, Anti-integrin therapy, Anti-IL-23 therapy, JAK inhibitor, Other biologic/targeted therapy, specify, NONE
Total # hospitalizations for IBD flare (whole #)
Hospitalization or corticosteroids for IBD flare within past year? (YES / NO)
**ENDOSCOPIC ASSESSMENT VARIABLES**
Date of Endoscopic Assessment:
Active inflammatory disease (macroscopic) ? (YES / NO)
Extent of active inflammatory disease (macroscopic)?
Post-inflammatory polyps? NONE / FEW / MODERATE / SEVERE
Extensive scarring or colon foreshortening? (YES / NO)

Participant ID: ☐☐ ☐☐☐

Total procedure time (colonoscope entry to exit) __ (minutes) __ (seconds)
Total withdrawal time(cecum to rectum) __ (minutes) __ (seconds)
Total # non-targeted (random) biopsies throughout colon and rectum (i.e. any biopsy NOT from a suspicious lesion or its immediate periphery, whether inflamed or uninflamed)
Total # unique lesions or suspicious areas of mucosa areas that were biopsied
Endoscopic resection of polyp or flat lesion?
**NEW INTRA/PERI-PROCEDURAL ADVERSE EVENT (Day of Procedure)**
Complication requiring admission to hospital? (YES/NO)
Bleeding requiring hospital admission and/or blood transfusion? (YES/NO)
Bowel perforation? (YES/NO)
Cardiac or respiratory complication requiring admission to hospital? (YES/NO)
Other major procedural complication? (YES/NO) [If yes, please specify]
**NEW POST-PROCEDURAL ADVERSE EVENT (2 weeks Post Procedure)**
Date of Post-Procedural Adverse Event:
Admission to hospital (for any indication)? (YES / NO)

Emergency department visit (for any indication)? (YES / NO)
Procedure-related admission or ED visit? (YES / NO)
Severe rectal bleeding requiring hospital admission, blood transfusion or repeat colonoscopy? (YES / NO)
Bowel perforation? (YES / NO)
Cardiac or respiratory complication requiring hospital admission? (YES / NO)
Other significant complication? (YES/ NO) [If yes, please specify]
Death? (YES / NO)<br><br>[if yes, date of Death: DD/MMM/YYYY]
**HISTOLOGY VARIABLES**
Total # unique neoplastic foci from non-targeted (random) and targeted biopsies (N.B. each non-targeted biopsy or unique visible neoplastic lesion counts as a unique focus; multiple targeted biopsies of the same lesion are counted collectively as one focus)
# of unique foci that are indefinite for dysplasia
tubular or tubulovillous adenomas
serrated adenomas (traditional or sessile)

unique neoplastic foci that were visible as discrete lesion or abnormal area during endoscopy
unique neoplastic foci in random biopsies (each random biopsy with neoplasia counts as unique focus, even if from same general area of bowel)
Highest grade of pathology (low-grade dysplasia/ high-grade dysplasia/ invasive cancer/ no dysplasia)
**PARTICIPANT IMPACT VARIABLES**
Will neoplastic findings from colonoscopy (macroscopic or microscopic) change management (i.e. therapy, surveillance interval, surgery) ? (YES / NO)
Will neoplastic findings from colonoscopy (macroscopic or microscopic) lead to earlier subsequent surveillance exam? (YES / NO)
Will neoplastic findings from colonoscopy (macroscopic or microscopic) lead to surgical referral? (YES / NO)
What is the planned timing of the next surveillance colonoscopy (in years) (Whole Number)
**NEW PROTOCOL DEVIATIONS (list category of deviation and specify deviation)**
Date PI aware of Protocol Deviation (DD/MMM/YYYY)
REB Approval (Yes / No)
Participant Eligibility (Yes / No)
Primary intervention (Yes / No)
Other endoscopic practice? (Yes / No)
Pathology processing or interpretation (Yes / No)

Participant ID: | | | | | |

Participant follow-up interview (Yes / No)
